# Immunomodulatory properties of stem cells from human exfoliated deciduous teeth

**DOI:** 10.1186/scrt5

**Published:** 2010-03-15

**Authors:** Takayoshi Yamaza, Akiyama Kentaro, Chider Chen, Yi Liu, Yufang Shi, Stan Gronthos, Songlin Wang, Songtao Shi

**Affiliations:** 1 Center for Craniofacial Molecular Biology, University of Southern California School of Dentistry, 2250 Alcazar Street, CSA 103, Los Angeles, CA 90033, USA; 2 Department of Oral Anatomy and Cell Biology, Kyushu University Graduate School of Dental Science, Fukuoka 812-8582, Japan; 3 Department of Molecular Genetics, Microbiology and Immunology, Robert Wood Johnson Medical School, University of Medicine and Dentistry of New Jersey, 661 Hoes Lane, Piscataway, NJ 08854, USA; 4 Mesenchymal Stem Cell Group, Division of Haematology, Institute of Medical and Veterinary Science/Hanson Institute/CSCR, University of Adelaide, Frome Rd, Adelaide, SA 5000, Australia; 5 Salivary Gland Disease Center and the Molecular Laboratory for Gene Therapy & Tooth Regeneration, Capital Medical University School of Stomatology, Tian Tan Xi Li No.4, Beijing 100050, PR China

## Abstract

**Introduction:**

Stem cells from human exfoliated deciduous teeth (SHED) have been identified as a population of postnatal stem cells capable of differentiating into osteogenic and odontogenic cells, adipogenic cells, and neural cells. Herein we have characterized mesenchymal stem cell properties of SHED in comparison to human bone marrow mesenchymal stem cells (BMMSCs).

**Methods:**

We used *in vitro *stem cell analysis approaches, including flow cytometry, inductive differentiation, telomerase activity, and Western blot analysis to assess multipotent differentiation of SHED and *in vivo *implantation to assess tissue regeneration of SHED. In addition, we utilized systemic SHED transplantation to treat systemic lupus erythematosus (SLE)-like MRL/*lpr *mice.

**Results:**

We found that SHED are capable of differentiating into osteogenic and adipogenic cells, expressing mesenchymal surface molecules (STRO-1, CD146, SSEA4, CD73, CD105, and CD166), and activating multiple signaling pathways, including TGFβ, ERK, Akt, Wnt, and PDGF. Recently, BMMSCs were shown to possess an immunomodulatory function that leads to successful therapies for immune diseases. We examined the immunomodulatory properties of SHED in comparison to BMMSCs and found that SHED had significant effects on inhibiting T helper 17 (Th17) cells *in vitro*. Moreover, we found that SHED transplantation is capable of effectively reversing SLE-associated disorders in MRL/*lpr *mice. At the cellular level, SHED transplantation elevated the ratio of regulatory T cells (Tregs) via Th17 cells.

**Conclusions:**

These data suggest that SHED are an accessible and feasible mesenchymal stem cell source for treating immune disorders like SLE.

## Introduction

Human bone marrow mesenchymal stem cells (BMMSCs) have been identified as a population of postnatal stem cells with the potential to self-renew and differentiate into osteoblasts, chondrocytes, adipocytes, and neural cells [1-5]. BMMSCs also exhibit immunomodulatory and regulatory effects on T and B lymphocytes, dendritic cells, and natural killer cells, indicating an attractive feature for cell therapy [6-11]. In addition, culture expanded BMMSCs may fail to express MHC-class II antigens on their surfaces, therefore allogenic BMMSCs have been used in treating a variety of diseases such as acute graft-versus-host-disease (GVHD) [12-14], ameliorating Hematopoietic Stem Cell engraftment [15,16], and systemic lupus erythematosus (SLE) [17]. Recently, mesenchymal stem cells derived from other tissues have also been found to possess immunomodulatory functions [18-20] which offer opportunities to find more effective and feasible mesenchymal stem cell sources for cell therapies.

Stem cells from human exfoliated deciduous teeth (SHED) have been isolated from naturally exfoliated deciduous teeth with the capacity to differentiate into osteogenic and odontogenic cells, adipocytes, and neural cells [21]. As neural crest cell-associated postnatal stem cells, SHED express a variety of neural cell markers including nestin, beta III tubulin, GAD, NeuN, GFAP, NFM, and CNPase [21]. Also, SHED are able to form bone when transplanted *in vivo *[22] and offer obvious bone regeneration for repairing calvarial defects in a mouse model [23]. It is unknown whether SHED possess immunomodulatory function as seen in BMMSCs. In this study, we compare immuno-regulatory properties between SHED and BMMSCs and utilize SHED transplantation to treat SLE-like diseases in a murine model.

## Materials and methods

### Mice

C57BL/6J and C3MRL-Fas^*lpr*^/J (MRL/*lpr*) mice (female, six- to seven-week-old) were purchased from the Jackson Laboratory (Bar Harbor, ME, USA). Beige *nude*/*nude *Xid (III) mice (female, 8- to 12-week-old) were purchased from Harlan (Indianapolis, IN, USA). All animal experiments were performed under an institutionally approved protocol for the use of animal research (University of Southern California protocol #10874 and #10941).

### Human tooth, bone marrow and peripheral blood samples

Human exfoliated deciduous incisors were obtained as discarded biological samples from children (six- to eight-year-old) at the Dental Clinic of the University of Southern California following the approved Institutional Review Board guidelines. Healthy bone marrow aspirates from iliac bone and peripheral blood mononuclear cells (PBMNCs) of healthy volunteers were purchased from AllCells (Berkeley, CA, USA).

### Isolation and culture of SHED and BMMSCs

Mononuclear cells isolated from the remnant dental pulp tissue of the deciduous incisors were cultured as reported previously [21,24]. BMMSCs culture was described previously [25]. The detailed protocols were described in Additional file 1.

### Cell surface markers analysis

The procedure for single colored flow cytometry (FCM) was performed as described previously [[26], and Additional file 1]. The samples were analyzed on a FACS^Calibur ^flow cytometer (BD Bioscience, San Jose, CA, USA). Some cells were used for immunoblot analysis and immunofluorescent staining.

### Colony forming units-fibroblastic (CFU-F) assay

CFU-F assay was performed according to a previous study [[27], and Additional file 1].

### Cell proliferation assay

The proliferation of each MSC population was performed by bromodeoxyuridine (BrdU) incorporation assay as previously described [[21,27] and Additional file 1].

### Telomerase activity assay

Telomerase activity was evaluated by telomeric repeat amplification protocol (TRAP) assay using real-time polymerase chain reaction (PCR) [[28], and Additional file 1].

### *In vitro *osteogenic induction assay

Osteogenic differentiation assays of SHED and BMMSCs were performed according to previous publications [21,28]. Osteogenic markers and mineralized nodule formation were assessed as described previously [[21,28] and Additional file 1].

### Adipogenic induction assay in vitro

Adipogenic assay *in vitro *of each stem cell population was performed as described previously [[21,28], and Additional file 1].

### *In vivo *osteogenic differentiation

Xenogeneic transplantation was performed using immunocompromised mice as described [21,25,26]. Each MSC population was subcutaneously transplanted into beige *nude*/*nude *Xid (III) mice using hydroxyapatite tricalcium phosphate (HA/TCP) as a carrier. Eight weeks post-transplantation, the transplants were harvested for histological analysis. Detail methods were described in the Additional file 1.

### Immunoblot analysis

Ten μg total protein was loaded and analyzed by immunoblotting as previously described [[21,28], and Additional file 1].

### Co-culture of human PBMNCs or T lymphocytes with SHED or BMMSCs

PBMNCs or T cells were co-cultured with or without SHED or BMMSCs under several culture conditions as described in Additional file 1. Cell death analysis and induction of Tregs and Th17 cells were described in Additional file 1

### Xenogeneic SHED or human BMMSCs into MRL/*lpr *mice

Under general anesthesia, SHED or BMMSCs (1 × 10^5 ^cells/10 g body weight in 100 μl PBS) were infused into MRL/*lpr *mice via tail vein at 16 weeks (n = 3) according to previous study [17]. MRL/*lpr *mice (16-week-old) received physiological saline (n = 3) were used as experimentally control mice. All mice were sacrificed at 20 weeks of age, and from them were collected peripheral blood, kidney, and long bones (femur and tibiae).

### FCM analysis of Treg and Th17 cells

Flow cytometric staining and analysis were performed as previously reported [[29], and Additional file 1].

### Measurement of biomarkers in culture supernatant, blood serum and urine

Several biomarkers, including anti-dsDNA antibody and anti-nuclear antibody ANA, complement 3 (C3), interleukin 6 (IL6), IL10, IL17, soluble receptor activator for nuclear factor κB ligand (sRANKL), and C-terminal telopeptides of type I collagen (CTX), creatinine, urine protein in biofluid samples (peripheral blood serum and urine) were measured by enzyme linked immunosorbent assay (ELISA) [[17], and Additional file 1].

### Histological analysis of kidney and bone

Kidneys and long bones (femurs) harvested from mice were fixed and processed to make paraffin sections. The sections were used for further experiments [Additional file 1].

### Histometry

Histomorphometric analysis was quantified as described previously [25]. Detailed methods were described in Additional file 1.

### Statistics

All data are expressed as the mean ± SD of, at least, triplicate determinations. Statistical difference between the values was examined by Student's t-test. The *P *values less than 0.05 were considered significant.

### Antibodies and primers

All primary antibodies used in this study were described in Additional file 1 and listed on Table S1 in Additional file 1. All primer pairs were listed in Table S2 in Additional file 1

## Results

### SHED possess mesenchymal stem cell properties

**Figure 1:**
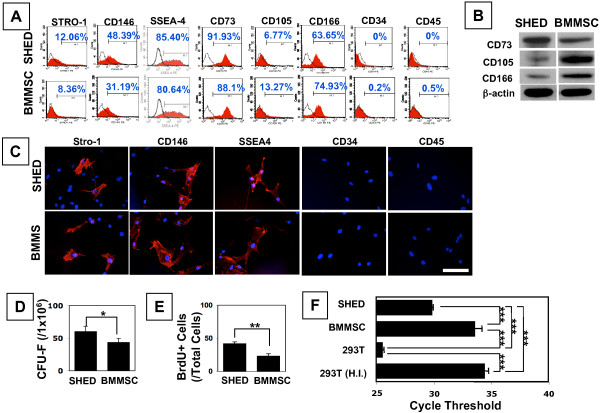
**Characterization of SHED in comparison to BMMSCs**. **(A) **Flow cytometric analysis of cultured SHED at passage 3 revealed expression of STRO-1 (12.06%), CD146 (48.39%), SSEA4 (85.40%), CD73 (91.93%), CD105 (6.77%), CD166 (63.65%), but was negative for surface molecules CD34 and CD45. SHED express high levels of STRO-1 and CD146 (n = 5; *P *< 0.05) and low level of CD105 (n = 5; *P *< 0.01) compared to expression levels of STRO-1 (8.36%), CD146 (31.19%), and CD105 (13.27%) in BMMSCs. These signals are shown as the red area. Solid lines indicate signals for isotype matched control antibodies. M1 window show the positive expression defined as the level of fluorescence greater than 99% of the corresponding isoytpe-matched control antibodies. Representative histograms are shown among five donors. (**B**) Immunoblot analysis confirmed expression of CD73, CD105 and CD166 in SHED and BMMSCs. Representative images of n = 5 donors are presented as results. (**C**) Immunofluoresence confirmed that SHED express STRO-1, CD146, and SSEA4 along with negative for CD34 and CD45. Red fluorescence indicates the expression of cell surface markers. Blue cell nuclei were stained by DAPI. Images were representative data of independent experiment (n = 5) with consistent results (Bar = 50 μm). (**D**) SHED were able to form significantly high numbers of single colonies than BMMSCs when 1 × 10^6 ^cells were plated at a low density (*P < 0.05) and cultured for 10 days. (**E**) The proliferation rates of SHED and BMMSCs were assessed by co-culture with BrdU for 18 hours. The number of BrdU-positive cells was presented as a percentage of the total number of cells counted from five replicate cultures. SHED showed a significantly higher proliferation rate in comparison to BMMSCs (**P < 0.01). (**F**) SHED showed a high activity of telomerase compared to BMMSCs assessed by real time PCR. HEK293T cells (239T) were used as a positive control and heat inactivated 293T (H.I.) cells were used as a negative control. The activity was indicated by a PCR cycle threshold and averaged from three replicated cultures (***P < 0.001).

Although SHED are capable of differentiating into a variety of cell types [21], their detailed mesenchymal stem cell properties remain to be elucidated. Herein, we used flow cytometry, immunoblot analysis, and immunocytostaining analysis to demonstrate that SHED at passage 3 expressed many mesenchymal surface markers, including STRO-1, SSEA4, CD73, CD105, CD146, and CD166 but were negative for CD34 and CD45 (Figures 1A-C). In comparison to BMMSCs, SHED expressed significantly higher levels of STRO-1 and CD146, and lower levels of CD105 (Figure 1A). Additionally, SHED showed significantly high numbers of single colony clusters (colony-forming units-fibroblastic; CFU-F) and an elevated cell proliferation rate compared to BMMSCs (Figures 1D and 1E). This elevated proliferative capacity may be associated with the significantly increased telomerase activity in SHED (Figure 1F).

**Figure 2:**
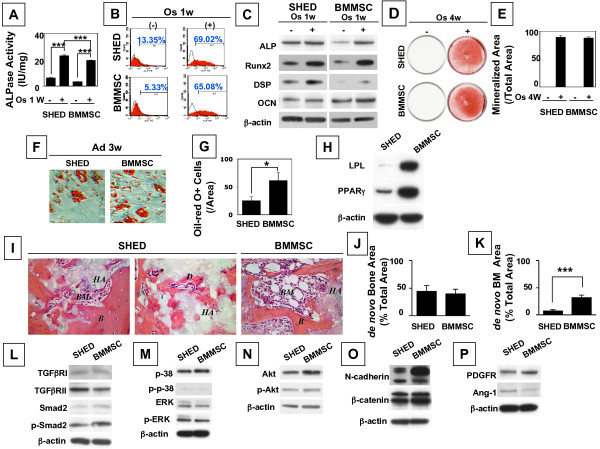
**Mesenchymal stem cell properties of SHED**. (**A-E**) SHED showed a similar osteogenic differentiation potential to BMMSCs. After one week culture induction under osteogenic conditions, ALP activity and numbers of ALP positive cells in SHED and BMMSCs were significantly higher than that of the control SHED and BMMSCs, respectively, by ALP staining (Representative of n = 5) (**A**) and flow cytometric analysis (Representative of n = 3) (**B**). Meanwhile, immunoblot analysis showed that the osteogenic induction elevates expression levels of ALP, Runx2, DSP, and OCN in SHED and BMMSCs (**C**) (***P < 0.001, n = 5). β-actin was used as an internal control. After four weeks culture induction in osteogenic medium, SHED showed increased capacity of forming mineralized nodules as assessed by alizarin red staining (Representative of n = 5). (**D**). Alizarin red-positive area corresponding to total area was averaged from five independent groups (**E**). (**F-H**) SHED showed reduced potential of differentiating into adipocytes compared to BMMSCs. Three weeks post adipogenic induction, lipid accumulation in SHED was less than that in BMMSCs by Oil-red O staining (Representative of n = 5). (**F**). Number of oil-red O-positive (Oil-Red-O+) cells was calculated as a percentage to total cells and averaged from five independent cultures (**G**) (*P < 0.05). Immunoblot assay indicated that SHED expressed lower levels of adipocyte-specific molecules LPL and PPARγ than BMMSCs at three weeks post adipogenic culture (**H**). Three independent assays showed the similar results. (**I-K**) SHED were capable of forming mineralized tissue when transplanted subcutaneously into immunocompromised mice using HA/TCP as a carrier (Representative of n = 3). (**I**). It appeared that SHED form similar amounts of mineralized tissue as seen in a BMMSC transplant (Representative of n = 3) (**I**, **J**), but they generated significantly less bone marrow elements than BMMSCs (**K**). Newly formed mineralized tissue and bone marrow areas were calculated as a percentage of the total area and averaged from three independent transplant assays (***P < 0.001). *B *= bone, *BM *= bone marrow, *C *=: connective tissue, *H *=: hydroxyapatite and tricalcium carrier. (**L-P**) SHED and BMMSCs express multiple signaling pathways during culture expansion at passage 3. SHED and BMMSCs expressed TGFβ receptor I and II, Smad 2 and phosphorylated Smad 2 (**L**); P38, phosphorylated P38, ERK, and phosphorylated ERK (**M**); Akt and phosphorylated Akt (**N**); N-cadherin and β-catenin (**O**); PDGF receptor and Ang-1 (**P**). Representative image of n = 5.

To compare osteogenic differentiation of SHED with BMMSCs, multiple colony-derived SHED at passage 3 were supplemented with L-ascorbate-2-phosphate, dexamethasone, and inorganic phosphate to induce mineralization *in vitro *as described previously [21]. After one week of induction, SHED were similar to BMMSCs, showing significantly increased alkaline phosphatase (ALP) activity (Figure 2A) and the number of ALP-positive cells by flow cytometric analysis (Figure 2B), and expression of elevated levels of ALP, Runt related transcription factor 2 (Runx2), dentin sialoprotein (DSP), and osteocalcin (OCN) by immunoblot analysis (Figure 2C). Alizarin Red-positive nodule formation in SHED and BMSMC cultures was notified after four weeks of osteogenic induction, indicating calcium accumulation *in vitro *(Figures 2D and 2E). However, SHED suffered remarkable impairment of adipogenic differentiation, as shown by decreased numbers of lipid-specific Oil red O-positive cells and reduced expression of adipocyte-specific molecules, peroxisome proliferator-activated receptor γ2 (PPARγ2) and lipoprotein lipase (LPL) when compared to BMMSCs (Figures 2F-H). To validate the capacity of forming mineralized tissue *in vivo *by SHED, *ex vivo *expanded-SHED were transplanted into immunocompromised mice with hydroxyapatite/tricalcium phosphate (HA/TCP) as a carrier. SHED formed a similar amount of mineralized tissue and a reduced amount of hematopoietic marrow components when compared to BMMSC transplants (Figures 2I-K). Next, we confirmed that SHED were similar to BMMSCs in activation of multiple signaling pathways, including TGFβ, ERK, Akt, Wnt, and PDGF (Figures 2L-P).

### Interplays between SHED and T-lymphocytes

**Figure 3:**
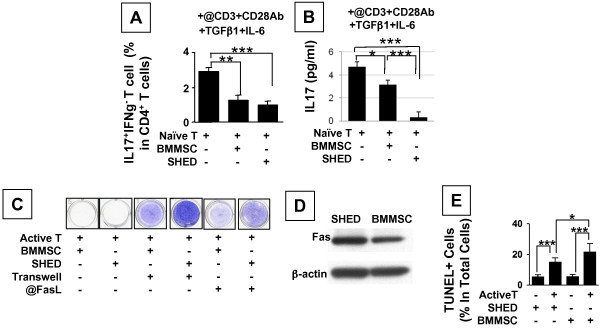
**SHED interplay with T-lymphocytes**. (**A, B**) Under the anti-CD3 and CD28 antibody along with TGFβ1 and IL-2 stimulation, SHED showed a significant effect in reducing Th17 cell levels as seen in BMMSCs (**A**), however, SHED exhibited a significant capacity of inhibiting IL17 levels than BMMSCs (**B**) (n = 3, *P < 0.05,***P < 0.001). **(C) **PBMNCs activated by anti-CD3 antibody (@CD3Ab, 1 μg/ml) were capable of inducing significant SHED and BMMSC death as shown by toulidin blue staining. When cells were cultured in an indirect co-culture system using Transwell, activated slpenocytes they failed to induce SHED and BMMSC death. Neutralization with anti-FasL antibody (@FasLAb, 1 μg/ml) blocked PBMNC-induced SHED and BMMSC death. Representative of n = 3. (**D**) SHED express a higher level of Fas in comparison to that in BMMSCs by immunoblotting. Three independent experiments showed similar results. Representative of n = 3. (**E**) SHED death caused by active PBMNCs is through an apoptotic pathway according to the TUNEL staining. The SHED death rate was similar to BMMSCs. The percentage of TUNEL-positive (TUNEL+) nuclei was indicated to the total number of MSCs and averaged from five replicated cultures (***P < 0.005).

In order to compare the immunomodulatory capacity of SHED with BMMSCs, anti-CD3/CD28 antibodies with TGFβ/IL-6 were added to the co-cultures of SHED or BMMSCs with naïve T cells, which were purified from human PBMNCs, levels of IL17^+^IFNg^- ^Th17 cells and IL17 were significantly reduced in SHED and BMMSC groups compared to the naïve T cell group (Figure 3A). It appeared that SHED showed a significant inhibiting effect in reducing IL17 levels when compared to BMMSCs (Figure 3B). Our previous report indicated that activated T cells induce apoptosis of BMMSCs through the Fas/FasL pathway [28]. To determine whether activated T cells also directly impinge on SHED, as occurs in BMMSCs, SHED were co-cultured with human PBMNCs activated by anti-CD3 specific antibody treatment. We found that the activated PBMNCs were able to induce part of SHED death in the co-culture system (Figure 3C). When SHED were separated from PBMNCs using a Transwell co-culture system or treated using anti-FasL neutralizing antibody, SHED failed to show the cell death (Figure 3C), suggesting that direct cell-cell contact and the Fas/FasL pathway are required for inducing SHED death by activated splenocytes. Next, we confirmed that SHED express Fas by immunoblot analysis (Figure 3D). Terminal deoxynucleotidyl transferase-mediated dUTP-biotin nick end labeling (TUNEL) staining was used to confirm that the SHED death was due to cell apoptosis (Figure 3E).

### SHED transplantation improves SLE phenotypes in MRL/*lpr *mice

**Figure 4:**
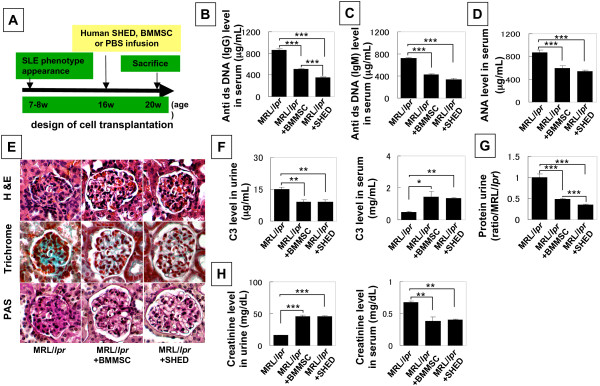
**SHED transplantation reduced levels of autoantibodies and improved renal function in MRL/*lpr *mice**. **Figure 4A **shows the scheme of SHED and BMMSC transplantation procedures. (**B-D**) ELISA quantified that levels of anti dsDNA IgG (**B**), IgM (**C**) and nuclear (**D**) antibodies (ANA) (mean ± SD) were significantly reduced in the peripheral blood of SHED and BMMSC treated MRL/*lpr *mice (n = 6) when compared to un-treated MRL/lpr mice c (n = 6) (***P < 0.001). It appeared that SHED transplantation resulted in a more significant reduction in anti IgG when compared to BMMSC transplantation (**B**). (**E**) MRL/*lpr *mice showed renal disorders such as nephritis with glomerular basal membrane disorder and mesangium cell over-growth. SHED and BMSSC transplantation resulted in a reduced basal membrane disorder and mesangium cell over-growth in glomerular (*G*) (upper panels, H&E staining; middle panels, trichrome staining; lower panels, periodic acid-schiff staining). Representative images of un-treated, SHED and BMMSC MRL/*lpr *(n = 6). (**F**) ELISA analysis showed that SHED transplantation has the same effect as seen in BMMSC transplantation in significantly reducing C3 level in urine and elevating C3 level in serum (n = 6, *P < 0.05, **P < 0.01). (**G**) SHED transplantation significantly reduced urine protein levels (mean ± SD) compared to BMMSC transplanted MRL/*lpr *mice (n = 6). (^[[[^*P *< 0.001). (**H**) Markedly increased urine creatinine and reduced serum creatinine were observed in SHED and BMMSC transplanted MRL/*lpr *mice (n = 6) compared to un-treated MRL/lpr mice (n = 6, ^[[[^*P *< 0.001, ^[[^*P *< 0.01).

Our previous study showed that systemic infusion of BMMSCs offers appropriate treatment for SLE disorders in human patients and SLE-like MRL/*lpr *mice [17]. Here we selected SLE-like mice at 16 weeks of age to infuse SHED for treating SLE disorders using BMMSCs as a control (Figure 4A). It is known that autoantibodies play a crucial role in SLE patients. Our previous study showed a remarkable increase in the levels of autoantibodies including anti-double strand DNA (dsDNA) IgG and IgM antibodies, and anti-nuclear antibody (ANA) in the peripheral blood [17]. As seen in BMMSC transplantation, SHED transplantation resulted in a significant reduction in serum levels of anti-dsDNA IgG and IgM, and ANA antibodies (Figures 4B-D).

Histological analysis with hematoxylin and eosin, trichrome, and periodic acid-Schiff staining revealed that SHED transplantation was similar to BMMSC transplantation in recovery of SLE-associated renal disorders, such as nephritis with glomerular basal membrane disorder and messangial proliferation in MRL/*lpr *mice (Figure 4E). ELISA data showed that SHED and BMMSC transplantation was able to reduce the urine C3 level and elevate the serum C3 level (Figure 4F). Also, SHED transplantation significantly reduced urine protein levels compared to BMMSC transplantation (Figure 4G). Moreover, SHED and BMMSC transplantation significantly elevated creatinine levels in urine and reduced creatinine levels in serum (Figure 4H). This experimental evidence indicated that SHED transplantation is an effective approach for treating SLE disorders.

### SHED transplantation regulates ratio of Tregs and Th17 cells

**Figure 5:**
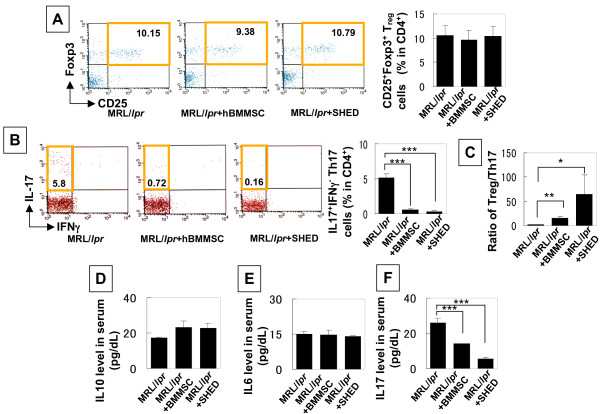
**The ratio of Tregs and Th17 cells may contribute to SHED mediated treatment in MRL/*lpr *mice**. (**A-C**) Flow cytometric analysis showed that the number of CD25^+^Foxp3^+ ^Tregs in CD4^+ ^T lymphocytes of MRL/*lpr *spleen was not significantly changed in SHED and BMMSC transplantation (**A**). In contrast, SHED and BMMSC transplantation were capable of significantly reduced levels of CD4^+^IL17^+ ^cells in spleen as compared to un-treated MRL/lpr mice (**B**). SHED transplantation significantly increased the ratio of Tregs and Th17 cells when compared to BMMSC transplantation group (**C**) (^[[[^*P *< 0.001, ^[[^*P *< 0.01, ^[^*P *< 0.05). Results were shown as mean ± SD from un-treated, SHED and BMMSC MRL/*lpr *(n = 6). (**D-F**) Although SHED and BMMSC transplantations failed to alter IL10 (**D**) and IL6 (**E**) levels in serum of MRL/lpr mice, IL17 levels were significantly down-regulated in SHED and BMMSC transplanted group compared to un-treated MRL/lpr mice (**F**). Results were shown as means ± SD from un-treated, SHED and BMMSC MRL/*lpr *(n = 6).

Tregs prevent pathogenic autoimmunity by suppressing proliferation and production of pro-inflammatory cytokines in effector immune cells, such as helper T-lymphocytes [30]. In contrast, Th17 cells that produce IL17 are inflammatory cells responsible for the pathogenesis of autoimmune diseases [31] and bone destruction [32]. Our previous study suggested that BMMSC transplantation affects the immune balance between Tregs and Th17 cells in SLE-like disorders [17]. Here we found that SHED transplantation showed more significant effect in up-regulating the ratio of Treg and Th17 cells in comparison to BMMSC transplantation in MRL/*lpr *mice (Figures 5A-C). Both SHED and BMMSC transplantations showed no significant changes in the level of IL10 and IL6 in MRL/*lpr *mice (Figures 5D and 5E); however, SHED transplantation provided a remarkable reduction of TH17 cells and IL17 level in MRL/*lpr *mice when compared to BMMSC transplantation (Figures 5C and 5F).

**Figure 6:**
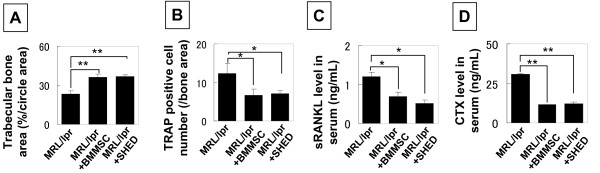
**SHED transplantation reconstructed trabecular bone and inhibited osteoclast activity**. (**A**) SHED transplantation showed the same effect in regenerating trabecular bone as seen in BMMSC transplanted MRL/*lpr *mice (n = 6) (^[[^*P *< 0.01). (**B**) TRAP staining showed that the number of TRAP positive osteoclasts was significantly reduced in SHED and BMMSC transplanted mice (n = 6, ^[^*P *< 0.05). (**C, D**) ELISA revealed that SHED and BMMSC transplantations were capable of significantly reducing the levels (mean ± SD) of soluble RANKL (sRANKL) (**C**) and C-terminal telopeptides of type I collagen (CTX) (**D**) in serum of MRL/*lpr *mice (n = 6)(*P < 0.05, **P < 0.01).

Our previous study suggested that BMMSC transplantation-mediated therapy in SLE-like mice may associate with the reconstructing trabecular bone [17]. Here we found SHED were also capable of reconstructing trabecular bone in MRL/*lpr *mice (Figure 6A). In contrast to BMMSC/osteoblast lineage, osteoclasts play a significant role in the maintenance of bone homeostasis by the bone resorption function. We compared SHED transplantation with BMMSC transplantation in inhibiting osteoclast activity in MRL/*lpr *mice and found that both SHED and BMMSC transplantation were able to reduce the number of tartrate-resistant acid phosphatase (TRAP)-positive osteoclasts in the distal femur epiphysis of MRL/*lpr *mice (Figure 6B), serum levels of runt-related NF-κB ligand (RANKL), a critical factor for osteoclastogenesis (Figure 6C), and bone resorption marker C-terminal telopeptides of type I collagen (CTX;) as compared to untreated MRL/*lpr *mice (Figure 6D).

## Discussion

BMMSCs have been successfully utilized to treat a variety of human diseases, such as bone fracture [33], severe aplastic anemia [34], acute GVHD [13], and SLE [17]. SLE is a common and potentially fatal immune disease in which autoantibodies damage multiple organs, including the kidneys, cardiovascular system, nervous system, joints, and skin [35]. The pathology of SLE involves the destruction of targeted organ tissues and accumulation of auto-reactive lymphocytes and immune complexes. Although intensity and organ involvement vary significantly among SLE patients, abnormalities of T and B lymphocytes are universal [35-37]. Moreover, SLE provokes multifaceted immune modulation, including both deficiency and hyperactivity of the immune system. An understanding of the underlying pathology is crucial to developing optimal therapies for the restoration of immune homeostasis without compromising the protective immune responses to pathogens [38]. MRL/*lpr *mice were generated by the insertion of the early transposable element ETn in the Fas gene, which causes a striking reduction in Fas mRNA expression and is associated clinically with marked acceleration of the lupus-like disease [39]. Levels of circulating immune complexes rise enormously from about three months of age in MRL-*lpr*/*lpr *but not in MRL mice. In this study, we used MRL/*lpr *mice as a SLE mouse model to indicate that SHED are an appropriate population of postnatal stem cells for SLE treatment as seen in BMMSC-mediated therapy.

SHED are derived from a very accessible tissue resource and capable of providing enough cells for potential clinical application via high proliferation rate and expression of telomerase [21]. The reason that SHED transplantation showed optimal therapeutic effect may be associated with the fact that SHED showed superior immunomodulatory effects compared to BMMSCs in terms of recovering Tregs/Th17 ratio and reducing Th17 cell levels in peripheral blood. In addition, SHED transplantation, as seen in BMMSC transplantation, is capable of recovering trabecular bone and inhibiting osteoclast activity, suggesting that SHED transplantation, as seen in BMMSC transplantation, could lead the reconstruction of osteoblastic niche to improve SLE disorders in SLE patients and a SLE-like murine model [17]. Therefore, SHED may be an appropriate stem cell resource for treating immune disorders via improved immunomodulatory properties. Systemic infusion of SHED fails to show a significant promoting Treg level in SLE-like mice as seen in an *in vitro *co-culture system, which may be associated with a complex *in vivo *condition that hardly compares to a simple co-culture system. However, SHED infusion resulted in a significantly up-regulated level of the ratio between Tregs and Th17 cells. This is an important index indicating immunomodulatory function of SHED due to the fact that Tregs prevent autoimmunity and Th17 cells promote autoimmunity and inflammation [40].

The transition from deciduous teeth to adult permanent teeth is a unique and dynamic process in which the development and eruption of permanent teeth is coordinated with the resorption of deciduous teeth. We found that exfoliated deciduous tooth crowns contain a remnant of living pulp comprised of a normal dental pulp structure, including connective tissue, blood vessels, and odontoblasts [21]. We demonstrated that these remnants of pulp tissues in exfoliated deciduous teeth contain SHED [21]. These studies provide the first evidence that a naturally occurring exfoliated organ contains stem cells with the ability to form multiple phenotypes, and that these stem cells may offer a unique stem cell resource for potential clinical applications. SHED are very easily acquired from exfoliated teeth and can be expanded *ex vivo *to achieve sufficient numbers of cells for tissue regeneration such as repairing parietal defects [24].

## Conclusions

SHED possess similar stem cell properties as those seen in BMMSCs, including osteo/odontogenic and adipogenic differentiation *in vitro*, forming mineralized tissue *in vivo*, and expression of extensive mesenchymal stem cell markers. Moreover, systemic SHED transplantation is capable of offering similar, if not better, therapeutic effect on SLE murine model, suggesting that easily accessed SHED may be a feasible stem cell source for stem cell therapies.

## Abbreviations

ALP: alkaline phosphatase; BMMSC: bone marrow mesenchymal stem cells; BrdU: bromodeoxyuridine; C3: complement 3; CFU: colony forming units-fibroblastic; CTX: C-terminal telopeptides of type I collagen; DSP: dentin sialoprotein; FCM: flow cytometry; HA/TCP: hydroxyapatite tricalcium phosphate; IL: interleukin; LPL: lipoprotein lipase; OCN: osteocalcin; PBMNCs: peripheral blood mononuclear cells; PCR: polymerase chain reaction; PPARγ2: peroxisome proliferator-activated receptor γ2; Runx2: Runt related transcription factor 2; Th17: T helper 17; TRAP: telomeric repeat amplification protocol; Tregs: regulatory T cells; TUNEL: Terminal deoxynucleotidyl transferase-mediated dUTP-biotin nick end labeling; SHED: Stem cells from human exfoliated deciduous teeth; SLE: systemic lupus erythematosus; sRANKL: soluble receptor activator for nuclear factor κB ligand.

## Competing interests

The authors declare that they have no competing interests.

## Authors' contributions

TY and KA collected and assembled data, and worked on data analysis and interpretation. CC collected and assembled data. YL worked on data analysis and interpretation. YS, SG and SW worked on conception and design, and SS worked on conception and design, data analysis and interpretation and wrote the manuscript.

## Supplementary Material

Additional file 1 **Supplementary Materials and methods and 2 supplementary tables**. A PDF file containing supplementary Materials and methods and 2 supplementary tables: Table S1 displays information on antibodies; and Table S2, lists PCR primers. Click here for file
