# Supplementary material for: Immunomodulatory properties of stem cells from human exfoliated deciduous teeth
Source: Stem Cell Res Ther. 2010 Mar 15;1(1):5. doi: 10.1186/scrt5 (PMC2873699; doi:10.1186/scrt5)
Supplement: Additional file 1 — Supplementary Materials and methods and 2 supplementary tables. A PDF file containing supplementary Materials and methods and 2 supplementary tables: Table S1 displays information on antibodies; and Table S2, lists PCR primers. [file scrt5-S1.PDF]

Supplementary Information

Immunomodulatory Properties of Stem Cells from Human Exfoliated Deciduous Teeth

TAKAYOSHI YAMAZA[a,b*], KENTARO AKIYAMA[a*], CHIDER CHEN[a], YI LIU[a], YUFANG SHI[c], STAN GRONTHOS[d], SONGLIN WANG[e], SONGTAO SHI[a]

[*]equal contribution

Materials and Methods

Isolation and Culture of SHED and BMMSCs. Minced remnant dental pulp tissue was digested in a fresh enzyme mixture. The enzyme solution contained 0.2% collagenase type I (Worthington Biochemicals Corp, Lakewood, NJ) and 0.1% dispase II (Roche Diagnostic/Boehringer Mannheim Corp., Indianapolis, IN) in phosphate buffered saline (PBS). After incubation for 60 min at 37°C, MNCs were obtained. Lymphocyte fraction was separated from bone marrow using a density gradient media Ficoll-Plaque™PLUS (GE Healthcare Bioscience, Piscataway, NJ). MNCs were passed through a 70-μm cell strainer (BD Bioscience, San Jose, CA). The single cell suspension of MNCs (1x10^{6}) was seeded on T-75 culture flasks (Corning, Corning, NY), and cultured at 37°C in 5% CO_2 in a growth medium. The growth medium contained alpha Modification of Eagle's Medium (αMEM; Invitrogen, Carlsbad, CA) supplemented with 15% fetal calf serum (Equitech-Bio Inc, Kerrville, TX), 100 μM L-ascorbic acid 2-phosphate (WAKO Pure Chemical Industries, Ltd., Osaka, Japan), 2 mM L-glutamine (Invitrogen), 100 U/ml penicillin and 100 μg/ml streptomycin (Invitrogen). After 3 hours, non-adherent cells were removed by washing with PBS, and the adherent cells were cultured. Colonies forming cells were recognized as stem cell clusters. The cells were passed and sub-cultured. For xenogenic stem cell transplantation (MSCT), SHED and BMMSCs were cultured under non-serum-depleted condition for 12 hours prior to MSCT, and repeatedly washed with physiological saline.

FCM for MSC cell surface antigen analysis. Passage 1 stem cells were cultured under the growth medium. Single-cell suspensions ($2x10^5$/100 μl each marker) were incubated with mouse monoclonal antibodies specific to cell surface markers (1 μg/100 μl each) for 45 min on ice, followed by reaction with R-phycoerythrin (PE) conjugated goat antibodies against mouse IgM or IgG (each 1 μg/100 μl, Southern Biotechnology) for 30 min on ice. As negative controls, isotype-matched mouse immunoglobulins (IgG_1, IgG_{2a} and IgM) (1 μg/100 μl each, Southern Biotechnology) were incubated instead of the primary antibodies. The cells were sorted on a $FACS^{Calibur}$ flow cytometer (BD Bioscience).

Immunofluorescent microscopy. The cells sub-cultured on 8-well chamber slides (Nunc, Rochester, NY) ($2x10^4$ per well) under the growth medium were fixed with 4% paraformaldehyde (PFA, Merck, Darmstadt, Germany), and blocked with normal serum matched to secondary antibodies. The samples were incubated with the specific antibodies to cell surface markers or isotype-matched mouse antibodies (1:50) overnight at 4^oC, and treated with Rhodamin-conjugated secondary antibodies (1:200, Jackson ImmunoResearch, West Grove, PA; Southern Biotechnology, Birmingham, AL). Finally, they were mounted by means of a Vectaseald mounting medium containing 4',6-diamidino-2-phenylindole (DAPI) (Vector Laboratories, Burlingame, CA).

CFU-F assay. MNCs ($10x10^3$ per flask) were seeded and incubated on T-25 culture flasks (Nunc) for 3 hours at 37^oC. The flasks were washed with PBS twice to remove non-adherent cells. Adherent cells on the flasks were cultured for 16 days in the growth medium, and stained with a fix/stain solution containing 0.1% toluidine blue (Merck) and 2% PFA (Merck) in PBS. Colonies containing >50 cells were recognized as single colony clusters under a microscope, and the colony numbers were counted.

BrdU incorporation assay. SHED and BMMSCs ($1x10^3$ per well) were seeded on 2-well chamber slides (Nunc) and cultured in the growth medium. After one to two days, BrdU reagent (1:100, Invitrogen) was added in the cultures. After 24 hours, incorporated BrdU was stained with the BrdU staining kit (Invitrogen), following the manufacture's instruction, followed by hematoxylin staining. To quantify proliferation capacity of the cells, ten representative images

were used to calculate BrdU-positive nuclei number. Cell proliferation capacity was shown as a percentage of BrdU-positive nuclei over total nucleated cells.

TRAP assay. To measure telomerase activity, TRAP assay was examined using the quantitative telomerase detection (QTD) kit (Allied Biotech, Inc., Ijamsville, MD) according to the manufactures' protocol. Briefly, P1 SHED or BMMSCs ($100x10^3$) cultured in the growth medium were mixed with 2xQTD pre-mix containing telomere primers (TTAGGG) and iQ™SYBR® Green Supermix (BioRad Laboratories, Hercules, CA), and detected with an iCycler iQ® real-time PCR Detection System (BioRad Laboratories). As positive control, HEK293T cells were used. The extracts were heated at 85°C for 10 min, and used as negative control. The real-time PCR condition was as follows: telomerase reaction for 20 min at 25°C, PCR initial activation step for 3 min at 95°C, 3-step cycling; denaturation for 10 sec at 95°C, annealing for 30 sec at 60°C, extension for 3 min at 72°C, and cycle number was 40.

Osteogenic induction assay *in vitro*. SHED or BMMSCs ($500x10^3$ per dish) were seeded on 100-mm dishes (Corning) and cultured in the growth medium until the cells reached at confluent condition. To induce osteogenic condition, the medium was changed to an osteogenic medium. The growth medium supplemented with 1.8 mM potassium dihydrogen phosphate (Sigma, St. Louis, MO) in the presence or absence of 10 nM dexamethasone (Sigma) was used as an osteogenic medium. One week after the osteogenic induction, osteogenic markers were analyzed by colormetry, FCM, and immunoblot analysis. To measure alkaline phosphatase (ALP) activity by colormetry, cultured MSCs were washed three times with PBS and collected for ALP activity analysis using Basic phospha-B test (Wako Pure Chemical) according manufactures' instruction and quantified the absorbance spectrophotometrically at OD_{405}. Total cellular protein was determined by using BCA protein assay kit (Pierce, Rockford, IL). ALP expression was also analyzed by FCM. Extracted total protein two weeks post osteogenic induction was used to analyze the expression of osteoblastic specific markers by immunoblotting. For mineralized nodule assay, cultured MSCs were stained with 1% alizarin red-S (Sigma) in distilled water at 4 weeks post induction. The alizarin red-positive area was analyzed using NIH image software Image-J and shown as a percentage of alizarin red-positive area over total area.

Adipogenic induction assay *in vitro*. Cells cultured until the confluent condition were induced in an adipogenic medium with the growth medium plus 500 μM isobutyl-methylxanthine (Sigma), 60 μM indomethacin (Sigma), 0.5 μM hydrocortisone (Sigma), and 10 μM insulin (Sigma) for three weeks. Some cultures were stained with 0.3% Oil-red-O (Sigma) to detect lipid droplets. The number of Oil-red O-positive droplets-containing cells were counted and shown as a percentage of Oil-red O-positive cells over total cells. Total protein was also extracted and analyzed adipocyte-specific markers by immunoblotting.

Osteogenic Differentiation *in vivo*. MSCs ($4.0x10^6$) were mixed with hydroxyapatite/tricalcium phosphate (HA/TCP) ceramic powders (40 mg, Zimmer Inc., Warsaw, IN). The mixture was implanted subcutaneously into the dorsal surface of 8-10-week-old beige *nude*/*nude* Xid (III) immunocompromised mice under the approved animal protocol of USC (#10874). The transplants were harvested 8 weeks after the implantation. For Histological analysis, the tissue samples were fixed with 4% PFA in PBS and decalcified with 5% EDTA solution (pH 7.4). The paraffin sections were stained with hematoxylin and eosin (H&E) and analyzed by ImageJ. Seven fields were selected and newly-formed mineralized tissue and newly formed bone marrow-like tissue area within each field was calculated and shown as a percentage of each tissue area over total tissue area.

Immunoblot assay. Cells were lysed in M-PER® mammalian protein extraction reagent (Pierce). Ten μg total protein was applied and separated on 4-12% NuPAGE® gel (Invitrogen) and transferred on Immobilon™-P membranes (Millipore Corporation, Bedford, MA). The membranes were blocked with 5% non-fat dry milk and 0.1% Tween 20 for 1 h, followed by incubation with the primary antibodies (1:100-1000 dilution) at 4°C overnight. They were treated with horseradish peroxidase-conjugated rabbit or mouse IgG (Santa Cruz) (1:10,000) for 1 h, enhanced with a SuperSignal® West Pico Chemiluminescent Substrate (Pierce), and exposured on BIOMAX MR films (Kodak, Rochester, NY).

Isolation and culture of naïve PBMSCs and naïve T lymphocytes.
Human PBMNCs purchased from All Cells (Buckley, CA) were used as naïve PBMNCs. Human $CD4^+CD25^-$ naïve T lymphocytes were purified by negative selection from naïve splenocytes

using a $CD4^{+}CD25^{+}$ regulatory T cell isolation kit (Miltenyi Biotec, Aubun, CA) according to the manufacture's instruction with MACS LD and LS columns (Miltenyi Biotec) and a magnetic separator MidiMACS (Miltenyi Biotec). The naïve PBMNCs and naïve T lymphocytes (each $1x10^{6}$ per well) were cultured on 24-well multi-plates (Corning) under complete medium. The complete medium contained Dulbecco's Modified Eagle Medium (DMEM, Lonza) supplemented with 10% heat-inactivated FBS, 2mM L-glutamine, 50 mM 2-mercaptoethanol, 100 U/ml penicillin and 100 μg/ml streptomycin.

Survival assay of SHED co-cultured with splenocytes. MSCs ($200x10^{3}$ per well) were plated in 24-well flat-bottom plates (Corning), and cultured for 3 days. Activated PBMNCs ($1x10^{6}$) were loaded directly on MSCs or indirectly interacted with MSCs using transwell system (Corning). The cells were co-cultured in DMEM-based medium in the absence or presence of anti-FasL antibody (1 μg/ml). After three days, the wells were washed by PBS and stained using a fix/staining solution containing 2% PFA and 2% toluidine blue or an ApopTag Peroxidase In Situ Apoptosis Detection kit (Millipore) to detect apoptotic cells. To quantify cell death, ten representative images were used for counting positive nuclei number. Apoptotic cells were shown as a percentage of positive nuclei over total nucleated cells.

Tregs and Th17 induction assay co-cultured with MSCs. $CD4^{+}CD25^{-}$ T-lymphocytes (naïve T lymphocytes, naïve T cells) ($1x10^{6}$ per well) were pre-cultured on 24-well multiplates under the complete medium in the presence of plate bounded anti-CD3ε antibody (5 μg/ml) and soluble anti-CD28 antibody (2 μg/ml) for 3 days. MSCs (SHED and BMMSCs) ($20x10^{3}$ per well) were also seeded on other plates and cultured under the growth medium for 3 days. MSC cultures were washed with complete medium twice. The activated T-lymphocytes ($1x10^{6}$ per well) were loaded on the MSC cultures, and co-cultured in the complete medium with or without recombinant human TFGβ1 (2 ng/ml) (R&D Systems) and recombinant human IL2 (2 ng/ml) (R&D Systems) or recombinant human TGFβ1 (2 ng/ml) and recombinant human IL6 (50 ng/ml) (R&D Systems) for Tregs or Th17 induction. After 3.5 days, floating cells and culture medium were collected and centrifuged. The cells were used to analyze Tregs and Th17 levels by flow cytometer, and the supernatant was used to measure IL10 level by enzyme-linked immunosorbent assay (ELISA).

Staining of Tregs and Th17 for flow cytometry. For Treg staining, cells ($1x10^6$) were treated with PerCP-conjugated anti-CD4, fluorescein isothiocyanate (FITC)-conjugated anti-CD8a, allophycocyanin (APC)-conjugated anti-CD25 antibodies (each 1 μg/ml) for 45 min on ice under shield. They were stained with R-phycoerythrin (PE)-conjugated anti-Foxp3 antibody (1 μg/ml) using a Foxp3 staining buffer kit (eBioscience, San Diego, CA) for cell fixation and permeabilization according to the manufacture's protocol. For Th17 staining, cells ($1x10^6$) were incubated with PerCP-conjugated anti-CD4, FITC-conjugated anti-CD8a, followed by the treatment with R-PE-conjugated anti-IL17 and APC-conjugated anti-IFNγ antibodies (each 1 μg/ml) using a Foxp3 staining buffer kit. The cells were then sorted on a $FACS^{Calibur}$ flow cytometer (BD Bioscience).

Measurement of bioactive factors. Culture supernatant was collected from the co-culture of SHED or BMMSCs with activated naïve T cells. Blood serum was obtained from the peripheral blood collected from the retro-orbital plexus of mice. Urine was also collected from mice. All of the samples were stored at -20°C until used and re-centrifuged before the application for ELISA. Anti-dsDNA IgG and IgM antibodies, ANA, C3, IL6, IL10, IL17, sRANKL and CTX were measured using commercial available kits (anti-dsDNA antibodies, ANA, albumin, C3, alpha diagnostic; IL6, IL10, IL17 and sRANKL, R&D Systems; CTX, Nordic Bioscience Diagnostics A/S) according to the manufactures' instructions. Creatinine levels were assayed using a commercial kit (R&D Systems). The urine protein concentration was measured using a Bio-Rad Protein Assay (Bio-Rad). The results were averaged in each group. The intra-group differences were calculated between the mean values.

Semi-quantitative RT-PCR. Total RNA was isolated from the cultures using SV total RNA isolation kit (Promega) and digested with DNase I following the manufacture's protocols. The cDNA was synthesized from 100 ng of total RNA using Superscript III (Invitrogen). And then, PCR was performed using gene specific primers and Platinum PCR Supermix (Invitrogen). The amplified PCR products were subjected to 2% agarose gels which contain ethidium bromide and visualized by UV fluorescent. The intensity of bands was measured by using NIH image-J software and normalized to GAPDH. RT-PCR was repeated in five or six independent samples.

Histological analysis of kidney. Samples were fixed with 4% PFA for 24 hours at 4°C, and embedded with paraffin. Paraffin Sections were used for H&E staining, trichrome staining, and Periodic Acid Schiff (PAS) staining.

Mineralized tissue analysis. Femurs and transplant tissue were fixed with 4% paraformaldehyde (PFA) in phosphate buffered saline (PBS), pH 7.2, overnight at 4°C, and decalcified with 5% EDTA (pH 7.4) in PBS for 10 days at 4°C. Bone samples were dehydrated with a graduate series of ethanol, cleaned with xylen, and immersed in paraffin. The samples were embedded in paraffin and cut into 8-μm-thick sections. The sections were deparaffinized, rehydrated and used for H&E staining and further histochemical staining.

TRAP staining. Deparaffinized sections were re-fixed with a mixture of 50% ethanol and 50% acetone for 10 min. TRAP-staining solutions were freshly made (1.6% naphthol AS-BI phosphate in N, N-dimethylformamide and 0.14% fast red-violet LB diazonium salt, 0.097% tartaric acid and 0.04% $MgCl_2$ in 0.2 M sodium acetate buffer, pH 5.0) and mixed in 1:10. The sections were incubated in the solution for 10 min at 37°C under shield and counterstained with toluidine blue. All reagents for TRAP staining were purchased from Sigma.

Histomorphomety. Area of trabecular bone and bone marrow was measured on H&E stained slides. To quantify osteoclast activity in the bones, number of mature osteoclasts was determined by TRAP positive cells that attached on the bone surface. Osteoblastic niche was quantified by the number of osteoblasts lining on the bone surface per bone marrow area with H&E staining. Quantification of newly-formed bone and marrow area was measured on transplant sections with H&E staining. The number of cells and the area were measured from five to seven representative images each sample using an NIH Image-J. The data were averaged the means in each experimental group. The results were shown as each indicated percentage.

Supplementary Table 1. Information of Antibodies

Antibodies	Application	Antigen	Host	Subclass	Vender
Actin, β-	IB	Hu	Ms	IgG_1	Sigma-Aldrich
Akt	IB	Ms	Rb	IgG	Cell Signaling
Akt, phospho	IB	Ms	Rb	IgG	Cell Signaling
Alkaline phosphatase (ALP) (LF47)	FCM, IB	Hu	Rb	IgG	Dr. Larry Fisher
Angiopoietin-1 (Ang-1)	IB	Hu	Rb	IgG	Santa Cruz
Cadherin, N-	IB	Hu	Rb	IgG	IBL
Catenin, β-	IB	Hu	Rb	IgG	Sigma-Aldrich
CD3	CC	Hu	Ms	IgG_{2a}	BD Bioscience
CD4, PerCP-conjugated	FCM	Hu	Ms	IgG_1	eBioscience
CD4, PerCP-conjugated	FCM	Ms	Rt	IgG_{2a}	BD Bioscience
CD8a, FITC-conjugated	FCM	Hu	Ms	IgG_1	eBioscience
CD8a, FITC-conjugated	FCM	Ms	Rt	IgG_{2a}	BD Bioscience
CD25, APC-conjugated	FCM	Hu	Ms	IgG_1	eBioscience
CD25, APC-conjugated	FCM	Ms	Rt	IgG_1	BD Bioscience
CD28	CC	Hu	Ms	IgG_1	eBioscience
CD34	FCM, IF	Hu	Ms	IgG_1	BD Bioscience
CD45	FCM, IF	Hu	Ms	IgG_1	BD Bioscience
CD73, R-PE-conjugated	FCM, IB	Hu	Ms	IgG_1	BD Bioscience
CD105	FCM, IB	Hu	Ms	IgG_1	BD Bioscience
CD146, R-PE-conjugated	FCM, IB, IF	Hu	Ms	IgG_1	BD Bioscience
CD166, R-PE-conjugated	FCM, IB	Hu	Ms	IgG_1	BD Bioscience
Dentin Sialoprotein (DSP) (LF-21)	IB	Ms	Ms	IgG	Dr. Larry Fisher
Fas (CD95)	IB	Hu	Rb	IgG	Santa Cruz
Fas ligand (FasL)	CC	Ms	Hm	IgG	eBioscience
Forkhead box P3 (Foxp3), R-PR-conjugated	FCM	Hu	Ms	IgG_{2a}	eBioscience
Foxp3, R-PR-conjugated	FCM	Ms	Rt	IgG_{2a}	eBioscience
Lipoprotein lipase (LPL)	IB	Ms	Rb	IgG	Santa Cruz
IFNγ, APC-conjugated	FCM	Hu	Ms	IgG_1	eBioscience
IFNγ, APC-conjugated	FCM	Ms	Rt	IgG_{2a}	eBioscience
Interleuikin 17 (IL17), R-PR-conjugated	FCM	Hu	Ms	IgG_1	eBioscience
IL17, R-PR-conjugated	FCM	Ms	Rt	IgG_1	BD Bioscience
Osteocalcin (OCN) (LF32)	IB	Hu	Rb	IgG	Dr. Larry Fisher
p38	IB	Hu	Rb	IgG	Cell Signaling
p38, phospho-	IB	Hu	Rb	IgG	Cell Signaling
p44/42 (ERK1/2)	IB	Rt	Rb	IgG	Cell Signalung
p44/42 (ERK1/2), phospho-	IB	Hu	Rb	IgG	Cell Signaling
Peroxisome proliferators-activated receptor-gamma (PPARγ)	IB	Hu	Rb	IgG	Santa Cruz
Platelet derived growth factor receptor-β (PDGFRβ)	IB	Hu	Rb	IgG	Santa Cruz
Runx2	IB	Hu	Rb	IgG	Oncogene
Smad2	IB	Hu	Rb	IgG	Zymed
Smad2, phospho-	IB	Hu	Rb	IgG	Cell Signaling
Stage-specific embryonic antigen 4 (SSEA-4)	FCM, IF	Hu	Rb	IgG_3	Chemicon

Supplementary Table 2. PCR Primers

Gene Name	Sense	Antisense	GenBank Accession #	Product Size (base pair)
mouse RORγt	5’-CCGCTGAGAGGGCTTCAC-3’	5’-TGCAGGAGTAGGCCACATTACA -3’	AJ132394	230
mouse IL17	5’-GGGCTCTGCCTGAGTTGTAG-3’	5’-AGAAATTTCGAAGGCCTGGT-3’	NM_008509	198
mouse GAPDH	5’-CACCATGGAGAAGGCCGGGGG-3’	5’-GACGGACACATTGGGGGTAG-3’	NM_008084	418
